# Older patients’ opportunity to die at home: a qualitative study on home care nurses’ experiences

**DOI:** 10.1186/s12912-026-04530-1

**Published:** 2026-03-11

**Authors:** Nina Berntsen, Sidsel Ellingsen

**Affiliations:** 1 The Dignity Centre, Ulriksdal 10, Bergen, N-5009 Norway; 2 Faculty of Health Sciences, VID Specialized University, Ulriksdal 10, Bergen, N-5009 Norway

**Keywords:** Palliative care, Older patients, Elderly, Home care nursing, Dying at home, Frequent flyer, Age discrimination

## Abstract

**Aim:**

This study explores home care nurses’ experiences of facilitating home death for older patients.

**Background:**

In Norway, the number of individuals over 80 years is expected to increase by over 50% by 2030, leading to a heightened demand for health services for older patients with complex care needs. Despite many seniors wishing to die at home, the percentage who do so remains low; only 1.3% of those over 90 years died at home in 2023. A similarly low rate was observed among those aged 65–79, where 5.7% died at home.

**Method:**

A qualitative design with three focus groups and one individual interview with home care nurses using Braun and Clarke’s thematic analysis framework was conducted. To provide rigor in the study, the COREQ guidelines were followed.

**Results:**

Participants reported that planning for home death for older adults was rare; when the patients’ health deteriorated, they were often hospitalized and received aggressive treatment instead of receiving palliative care at home. Variations in services across municipalities affected the availability of care. Nurses frequently expressed that they felt alone, particularly during evenings and weekends, highlighting the crucial role of involving general practitioners in ensuring safe care.

**Conclusion:**

The findings indicate that home care nurses need increased competence on how to facilitate home death, better organization of health services, increased patient involvement, and enhanced collaboration between healthcare services to facilitate the possibility of more home deaths among the older patients.

**Clinical trial number:**

Not applicable.

## Introduction

While a significant majority prefer to die at home, institutional settings often remain the site of many deaths [1]. Most deaths do not occur suddenly or unexpectedly; an extended period of severe illness allows patients, families, and healthcare providers to plan for end-of-life care [1]. In Norway, the number of individuals aged 80 and above is projected to increase by over 50% by 2030, reflecting a broader trend of an aging population [2]. Many older individuals are living longer with advanced chronic diseases, resulting in complex health profiles that demand comprehensive healthcare services [3]. The Norwegian Directorate of Health’s white paper emphasizes that patients must be given greater freedom of choice regarding where to receive palliative care and where to die [4].

In 2023, there where14% home deaths in Norway. Of the 65–79 years age group, 5.7% died at home; of those 80–90 years of age, 4.5% died at home; and of those over 90 years old, only 1.3% died at home. Among old patients 70 and older, almost 50% died in nursing homes in 2023 [5]. For older patients over 80 with significant care needs, home death is rarely planned [6].

‘Elderly’ is defined as individuals aged 67 and above who are considered non-working. Government authorities in Norway use the term to refer to those aged 65 to 75, with distinctions made for those over 75 as ‘old’ and those over 85 as ‘very old’ [7]. In recent decades, the role of nurses in home healthcare has undergone a significant transformation in Norway, as a growing number of patients are requiring palliative care in their homes [8]. Nurses frequently collaborate with patients over the age of 75 who express a desire to end their lives at home [9]. However, most older individuals who require substantial care still predominantly receive it in institutional settings. As the preference for home deaths continues to rise, home care nurses (HCNs) will increasingly take on a role in facilitating this in the future [10].

## Background

Patients receiving end-of-life care at home were more likely to die at home [11].

A UK study has demonstrated the central role of community nurses in the provision of palliative care. The study further showed that providing palliative care in the community is experienced as both professionally rewarding and emotionally demanding, and that integrating palliative care into a generalist nursing role is challenging within the constraints of limited time and resources [12]

Patients with non-cancer diagnoses generally receive less palliative care than cancer patients, including shorter durations, partly because research and service models have focused on cancer diseases. Less predictable disease trajectories and difficulties identifying the terminal phase contribute to this inequity [13].

However, a Belgian nationwide study of 23,670 home-dwelling older adults who died with dementia showed that fewer than one quarter received palliative home care during the last two years of life, and that initiation often occurred very late in the disease trajectory. Use of palliative home care was associated with more appropriate end-of-life care and lower healthcare costs in the final weeks of life compared with no such support [14].

Non-cancer patients often have multiple chronic conditions with complicated disease trajectories and unpredictable prognoses [15]. Masumoto et al. [15] found that few patients die at home without care and support from HCNs.

International research has consistently shown that timely initiation of home healthcare, continuity of care, and future care planning are crucial for enabling home death, particularly for older patients with complex and unpredictable illness trajectories.

HCNs play a critical role in planning for home death [16]. Kjellstadli et al. [17] reported that patients were less likely to die at home if home care began late during their course of illness, likely due to the time required to build trust and rapport among nurses, patients, and family members. Furthermore, cancer patients comprise the largest patient group who die at home, likely due to receiving consistent support in the form of conversations and planning throughout their illness [17].

Home healthcare is vital in facilitating home death. Kjellstadli et al. [17] found that chances of dying at home improved when patients received increased assistance and continuity of care from home healthcare servicesMeanwhile, Ervik et al. [18] found that effective palliative care for patients living at home allows them to spend more time at home and increases their likelihood of dying at home.

HCNs have reported difficulties related to complex and disorganized systems and structures, highlighting the benefits of providing care in patients’ homes [19]. In a study examining planned home deaths, Sørstrøm et al. (2023) found that HCNs go to great lengths to enable home deaths, compensating for a system that is not robust enough. Nurses navigate barriers during the planning of home death, such as inadequate staffing. As a result, they often perform more tasks than expected, facing a constant dilemma between the ideal standard of care they envision and what they can provide with limited resources [20].

A study conducted in Norway found that nurses’ experiences with home death varied in both the incidents of planned home death and the culture among HCNs. Some nurses had limited experience with home death, which led to a lack of knowledge that affected their confidence when managing patients with complex palliative needs and support in end-of-life care situations. They also found that the shortage of nurses, the lack of medical supplies, and night shifts challenges affected whether they managed to help patients who wanted to die at home [16]. In Norway, the number of patients who die at home is relatively low, despite most preferring to do so [21]. As old patients become weakened by illness, their perception of their ability to remain at home may shift, potentially affecting their desire to stay at home as long as possible and to die there. Nurses must consider these perspectives in home healthcare [22]. End-of-life care for old patients at home is perceived to have higher quality and value than similar care in institutions [23]. Many old patients experience cognitive impairment, complicating their preferences for where they wish to die. In addition, home death is more feasible when families strongly desire it. Early discussions regarding cognitive impairment are essential for allowing patients to express their wishes [15].

Older individuals living with multimorbidity are expected to become the primary recipients of palliative care in the coming decades [24]. Patients with non-cancer diagnoses often have limited access to palliative care due to less predictable disease trajectories and challenges in identifying the terminal phase. Evidence shows that these patients receive less palliative care than those with cancer, including shorter durations of care, partly because research and service models have focused on single diseases and age-related structural inequalities have hindered access to care [13]. In particular, the connection between age discrimination and frailty is a barrier to the utilization of healthcare services [25] Patients who reported age discrimination were often frail compared to patients who did not report age discrimination. Significant delays in emergency care are more considerable for older persons, and they are more likely to be misdiagnosed due to age-related stereotypes from health professionals [25]. Age discrimination can also significantly increase the risk of frailty progression. Poor health-seeking behaviour can lead to further health decline [25]. In this context, a patient is defined as a revolving door patient if admitted to hospitals at least four times yearly. Patients who are revolving door patients are more likely to experience complications; they are twice as likely to be readmitted, often due to comorbidities. Old patients are more often revolving door patients, but most causes of admission could be prevented or modified, such as lack of follow-up, inadequate knowledge, and premature discharge [26]. Older patients are often not provided with future care planning, leading to increased anxiety and uncertainty about what lies ahead [27].

Studies have found that the likelihood of home death increased when home care services had ample capacity and funding. The chances of dying at home versus in the hospital were lower in municipalities with populations between 10,000 and 50,000 than in the smallest municipalities with fewer than 5000 residents. Additionally, women were less likely to die at home than men, and this likelihood decreased with age. However, individuals aged 89 to 90 had a lower probability of home death compared to hospital death than those aged 90 and older. In contrast, the opposite was observed for deaths occurring in nursing homes [21].

Previous research on home death has often only mentioned older patients in passing, indicating a gap that necessitates further investigation into the experiences of the nurses caring for these individuals. The government in Norway recommends that everyone should be able to live at home as long as possible and die at home if they wish to [2]. Ervik et al. [18] have also highlighted the need for more research on palliative care in community settings to establish a robust knowledge base.

## The study

### Aim

This study aims to explore HCNs’ experiences facilitating home deaths for old patients. It describes nurses’ experiences in this context, answering the following research question: What experiences do home care nurses have in facilitating home deaths for older patients?

### Study design

This study uses a qualitative design to explore, and interpret various experiences from home care nurses. The categorization of realities and deeper meanings is central to such studies [28]. Data were collected through three group interviews and one individual interview. Group and individual interviews can contribute to describe the participants’ experiences by gathering subjective and narrative data [28]. Group interviews were chosen as the primary method of data collection, as this approach facilitates interaction and discussion among participants, allowing for the exploration of multiple perspectives and the generation of rich and nuanced data. Given that data collection was conducted across four municipalities in Norway, group interviews were also a pragmatic choice, enabling the inclusion of several participants within a single interview. In one municipality, however, an individual interview was conducted at the request of the local administration, as resource constraints made it difficult to recruit a sufficient number of participants for a group interview. This alternative approach was considered appropriate, as the municipality was relatively large and the interview was expected to provide a valuable contribution to the overall dataset [29].

In the reflexive approach to thematic analysis, the researcher plays a core role and is actively involved in generating knowledge. The codes reflect the researcher’s interpretations of recurring themes and meanings found within the dataset. Themes do not passively emerge from the data or the coding process but are viewed as a manifestation of the researcher’s interpretive engagement with the data [30].

Both authors of this study are nurses. The first author is a registered nurse with advanced training in oncology nursing and extensive clinical experience in home care and nursing for older adults. The co-author is a certified nurse anaesthetist and a professor of nursing science, with substantial experience in conducting qualitative research.

While the first author had limited prior experience with qualitative research, the co-author contributed extensive methodological expertise. Throughout the research process, the authors held regular meetings to support ongoing discussion, reflexivity, and methodological rigor.

### Study setting

**Fig. 1:**
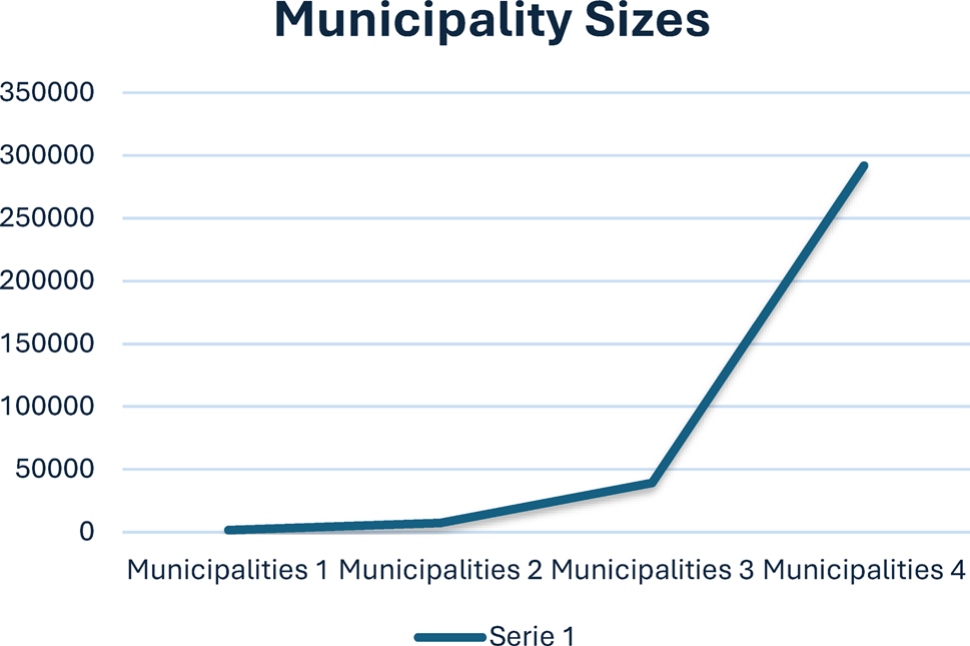
Sizes of the participating municipalities. (Number of inhabitants in each municipality.)

This study was conducted in four municipalities in Norway, one in the northern part and three in the western part. A Norwegian municipality is a local government unit responsible for providing public services and governing a specific geographic area. Each city or district has its administrative structure and budget, allowing it to address the unique needs of its community [31]. HCNs were recruited from both rural and urban municipalities, and the sizes of the participating municipalities varied substantially (Fig. 1).

### Participants and recruitment

Several municipalities were contacted regarding the possibility of recruiting participants, and four municipalities agreed to participate. Due to the different structures of home healthcare services across municipalities, there were different contacts for each municipality. In one municipality, a professional manager was contacted; in another, the health and care manager was contacted; in the third, a resource nurse was contacted; in the fourth, the interview was arranged through the municipality. All the municipalities provided valuable support in organizing the interviews and in identifying nurses with experience in handling home deaths. A contact person was established in each municipality to invite participants to the interview. The authors had no influence on this process other than liaising with the relevant contact persons. All study participants were nurses working in home healthcare.

Four participants were planned for each focus group interview, but only three attended due to illness and limited workplace resources. One individual interview was agreed upon, as it could be valuable for the study, given that it involved a large municipality compared to the others. All the participants were given information and consent forms in advance.

### Inclusion and exclusion criteria

This study has the following inclusion criteria: at least five years of experience in home care services and relevant experience in facilitating home death. Nurses with limited experience were planned to be excluded, and this information was included in the information provided to potential interview participants. However, a nurse with three years of experience in home nursing was included in the study, as she had several relevant experiences with home deaths that could provide valuable information for the study. The participants characteristics are presented in Table 1.

**Table 1:** Characteristics of the participants (*N* = 10)

Participant (HCN)	Age	Years working in home healthcare	Job
HCN 1	26	3	Nurse
HCN 2	34	6	Nurse
HCN 3	36	11	Palliative nurse
HCN 4	50	6	Nurse
HCN 5	50	13	Palliative nurse
HCN 6	50	17	Health leader
HCN 7	50	14	Oncology nurse
HCN 8	55	18	Palliative nurse
HCN 9	56	32	Oncology nurse
HCN 10	58	34	Oncology nurse

### Participants

The participants were all women.

## Data collection

Data collection took place from October 2024 to December 2024. The first author conducted all the interviews. A semi-structured interview guide with predefined themes was developed (Table 2). A colleague of the first author was the co-moderator in two of the interviews. There was a long-distance issue during one interview, and no co-moderator was present. In the individual interview, it was beneficial to the interview participant for only one interviewer to participate.

**Table 2:** Interview guide

Interview guide:
1. Experiences on facilitating home death for older patients - Do you have experience in discussing with patients where they wish to die? - Can you describe a situation in which a patient was able to die at home? What made that possible (approximately how old was the patient)? - Can you describe a situation where you felt you made a significant difference for a patient in the final stages of life? - What barriers prevent this from happening?
2. What can be done differently to enable home death for older patients who wish for it? - Have you considered what could have been done differently when home death was not achieved?
3. Can you describe your experiences collaborating with family members (and volunteers) when facilitating home death for elderly patients? -Have you received feedback from patients or family members about what could be improved surrounding home death?

### Interviews

A pilot interview was conducted with six participants who were colleagues of the first author; they all had a nursing background. This provided valuable feedback and reflections that were incorporated into the interview guide and the formal interviews.

The interviews were conducted in suitable locations: three in the home care nurses’ facility and one in a hotel with a nice conference room. Coffee and snacks were provided for the interview participants. All three group interviews and the individual interview were conducted in a semi-structured manner, with the topic of this study as the agenda. This interview method allows for flexibility in exploring the participants’ experiences. Each interview lasted approximately one hour.

All interviews commenced with the question, ‘What are your experiences with home deaths for older patients?’ This was followed by deeper inquiries regarding specific instances, particularly to elicit relevant examples from the participants. If any of the mentioned experiences warranted further exploration, follow-up questions were utilized to gain additional insights.

All the interviews were recorded on audiotape and the online Dictaphone app. Important observations made during the interviews were also noted, and the first author’s thoughts were recorded immediately afterward. We considered the dataset sufficient to address the research question, guided by the concept of information power [32]. Given the specificity of the study aim, the richness of the dialogue with participants, and the in-depth analysis conducted, we judged that the sample provided an appropriate basis for exploring the topic, while acknowledging that alternative perspectives may exist.

### Transcription

All the interviews were transcribed verbatim by the first author within a week to ensure that valuable information would not be lost. The data in this study consisted of the transcribed material from all four interviews: 67 pages with 34,062 words. This process resulted in a good overview of the data. All information that could be linked to the participants, such as their names, was carefully removed. Numbers one, two, and three, as well as various colours in the data material, were used to differentiate who said what.

### Data analysis

The data were analysed using Braun and Clarke’s [33] thematic analysis, which consists of six phases. This is an inductive approach where one systematically works through the data, identifying different parts of the data that appear interesting, relevant, or meaningful.

Phase one focuses on becoming intimately familiar with the data. This phase began with transcription, which was conducted by the first author, and continued through repeated readings of the transcripts. During this process, initial analytical ideas and reflections were noted in relation to the data [33]. The co-author was also involved at this stage by reading through the transcripts and initiating the coding process, thereby contributing to the early analytical development.

Phase two involves systematically working through the data to identify meaning units that appear to be potentially interesting, relevant, or meaningful to the research question, labelling them with codes, analytically meaningful descriptions [33]. The created codes was a condensate of the content of the meaning units. were straightforward and meaningful, designed to easy to recognize the contents. The authors discussed the codes many times during this process. Once all the data were coded, the codes – totalling 323 – were carefully reviewed several times and grouped.

**Fig. 2:**
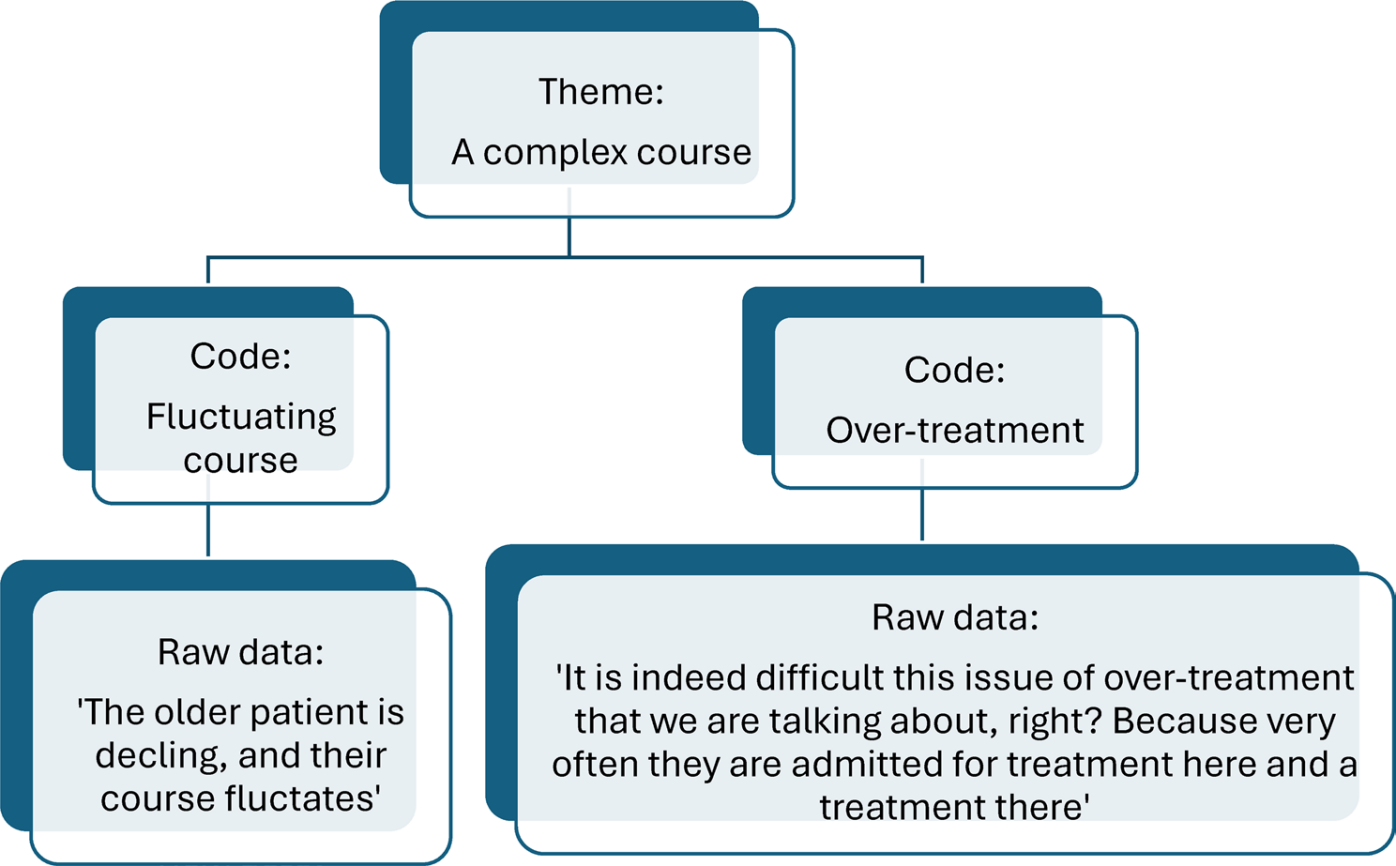
Example of the analysis process

In phase three, the objective is to identify patterns within the data. The researchers collect codes encapsulating similar main ideas or concepts while continuously reflecting on how these patterns can contribute to answering the research question [33]. The first author grouped these into preliminary themes, which were then discussed and thoroughly reflected on with the co-author. Fourteen overarching themes were identified and assigned temporary names. Figure 2 illustrates an example of the analysis process.

Phase four involves returning to the entire dataset to see how the preliminary themes fit. The themes need to be checked to determine if they make sense to both the data and the codes. Do the themes convey a compelling and convincing narrative about a significant pattern within the dataset? Additionally, the researcher assesses whether some themes can be integrated with existing research and practice.

This phase involved both authors working together to define, assemble, and deconstruct the themes, which required considerable time both independently and collaboratively. The researcher must articulate the findings in a manner that resonates with readers, illuminating both the intrinsic meanings found in the data and the researcher’s interpretation of those meanings [33].

**Fig. 3:**
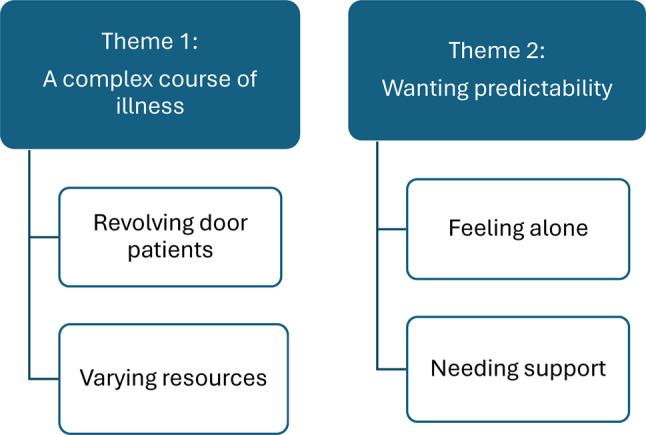
This figure presents an overview of the final themes and sub-themes

In phase five, the analysis is fine-tuned, ensuring that each theme is clearly articulated with a strong structure and essence. The interview data is continuously examined to convey the intended messages accurately. Throughout the analysis, both authors discussed and reflected together, engaging in collaborative deliberation. This effort led to refining and reviewing the themes [33], ultimately resulting in the two themes, presented in Fig. 3 with sub-themes and in the results section.

Phase six involves formulating the final results of the research. In an RTA project, the writing begins from the very start of the process, which is woven together in this phase. The four interviews provided a large and rich dataset, which necessitated a method to identify the codes and themes that would best address the research question in the analysis. Many data points have been saved for later use.

All the findings and quotations were translated from Norwegian into English, and the authors worked systematically to preserve the accuracy with which the study participants expressed themselves.

### Trustworthiness

The criteria of rigour, credibility, transferability, dependability, and confirmability were addressed [34]. Rigour is woven into the description of the data collection and the analysis. Credibility was maintained by the first author conducting all the interviews and the verbatim transcription, and it was further enhanced by repeated reflections and collaboration between the authors. Transferability was tried achieved by the description of the research process and findings, allowing readers to determine whether it can be transferred to their settings. Dependability was thoroughly addressed throughout the research process, with ongoing reflexivity regarding the researcher’s preconceptions. The first author continuously reflected on her prior understanding and experiences as a nurse in home health care, considering how these could influence the analysis. This prior knowledge was both a strength—allowing her to understand and contextualize the participants’ perspectives—and a potential limitation, as it could lead to premature conclusions if not carefully managed. Regular reflection throughout the study helped to mitigate this risk and supported a rigorous and balanced analytical process.) Confirmability was tried demonstrated by including participant quotations from the interviews to support the description of the findings.

Reflexivity is critical to rigour; as a qualitative researcher, it is essential to recognize the significance of self-awareness and reflexivity regarding one’s role in the process [34].

The first author’s clinical background and the second author’s extensive experience in qualitative research contributed substantially to the analytical process and overall methodological rigour of the study.

## Findings

### Theme 1: A complex course of illness

The nurses expressed satisfaction when they received the opportunity to contribute to the fulfilment of the patient’s wish to die at home. As HCN 10 stated, ‘It’s gratifying when you know they got their wish to die at home, that we managed to do it’. However, they had challenges in remembering when they had helped plan for home death for older patients. HCN 5 stated, ‘The older patient is declining, and their course fluctuates’.

The nurses described older patients’ conditions as gradually deteriorating over time, often accompanied by sudden fluctuations in health status. These fluctuations made it difficult to plan and anticipate the disease trajectory. In particular, the nurses felt that they were often involved too late in the course of the illness, which hindered their ability to establish relationships with the patient and their family. They also noted that the situation could become hectic and chaotic during the end-of-life phase for older patients.

#### Revolving door patients

The nurses reported that older patients frequently became revolving door patients at the end of their lives, meaning when their health condition deteriorated, they were repeatedly admitted to and discharged from the hospital during their final days. They also wondered if older patients truly wished to return repeatedly to hospital for treatment.

In one interview, the participants discussed a problematic patient situation:

- HCN 6:‘You often see that you have Plan A, but you also need Plan B, Plan C, and Plan D’.
- HCN 3:‘Yes, and it was likely fluid retention in one situation. I felt I couldn’t bear it when you started hearing those sounds. You can envision the scenario’.
- HCN 6:‘And if you’re standing there and can’t manage it, facing the choice of having a terrible experience at home or dying in an ambulance – that’s the decision we have to make’.
- HCN 3:‘It’s tough’.

The nurses often experienced unforeseen events that frequently occurred on Friday evenings when the general practitioner (GP) was unavailable, necessitating contact with emergency services. The emergency service, often unfamiliar with the patient, tended to readmit the older individual to the hospital, even when it had been decided that the patient should receive care at home if possible. For example, two informants described it this way during the interview:

- HCN 5: ‘Had we managed to identify the infection earlier, we could have asked if they wanted that, and what are we prolonging? Very often, they become revolving door patients towards the end’.
- HCN 7: ‘It isn’t easy. But I think this leads to fewer older patients dying at home’.

Another informant stated, ‘If the patient cannot be at home when life is nearing its end, they must go through emergency services. Unfortunately, we have no open wards’ (HCN 8).

The nurses emphasized the importance of having sufficient expertise to identify changes in health status early, which they believed was vital for allowing older patients to remain at home and die there. As HCN 3 stated, ‘In my experience, they will treat them almost to the point of death. Maintaining life. Setting boundaries for when enough is enough’.

#### Varying resources

The nurses worked in different municipalities, and it became clear that the available resources and the way home nursing was organized varied from place to place. Thus, it could seem arbitrary what assistance individual patients received at home. Some municipalities had reasonable arrangements and sufficient resources for older patients who wished to remain at home and die at home. In contrast, others lacked nighttime home care services, meaning that patients could not receive help at night if needed. In one municipality, they could hire a nurse to stay with the patient all night, allowing the relatives to rest. One nurse said: ‘When it’s possibly the beginning of the end, we hire a permanent caregiver. Whether it takes a week, two weeks, or three days, it takes as long as it takes’ (HCN 6). Meanwhile, a nurse from a different municipality commented on the possibility of hiring a permanent caregiver at night: ‘No. We can discuss the idea of a permanent caregiver, but your supervisors must be sure it’s the last night. It shouldn’t become a financial issue over five nights’ (HCN 7).

### Theme 2: Wanting predictability

The nurses found it challenging to ensure sufficient security to facilitate home deaths for older patients who wished to die at home. Often, they received a notification from the hospital on a Friday that the patient would be sent home on Saturday, with plans in place for the older individual to die at home. In the nurses’ experience, this could lead to what they described as chaos.

#### Feeling alone

The nurses often felt very alone while working in patients’ homes. They experienced a lack of confidence when faced with significant responsibilities regarding medications such as morphine; many were afraid of giving too much. This became even more difficult during evenings and weekends, when the emergency services were the only source of support available. They frequently found that older patients had various illnesses and symptoms, which made it particularly challenging for them to cope. Often, older patients were admitted to the hospital due to a lack of security in their homes.

One nurse described it this way: ‘We also have to feel secure as healthcare personnel today; we need to be confident in confronting and managing it at home. Perhaps more openness in staff groups is necessary to achieve that. We may unconsciously set certain boundaries without even realizing it’ (HCN 8).

#### Needing support

The GP was crucial in facilitating home deaths for older patients, but there were significant individual differences in GP support. The nurses felt a sense of security when the GP was involved, especially when they promptly responded to emails and visited patients at home. They felt substantially more secure when the GP provided their mobile number before a weekend when the dying patient might need extra support.

As one nurse said: ‘With home death, all these elements I’ve discussed must be planned, especially concerning general practitioners. We have more and more general practitioners willing to give out their mobile numbers. It provides great security for us and, not least, for the patient. We rarely use it, but at least we have it’ (HCN 7).

When an older patient was able to die at home, the nurses found that the patient usually had family members who felt secure in the situation. The family often managed to care for the dying individual themselves, and they might only need to make one or two calls to home care services at night. The nurses observed that men with a wife at home were more likely to die at home, as the presence of the wife provided a sense of security. As HCN 2 stated: ‘Yes, it’s the wife who has been the source of security and calm, right?’

### Ethical considerations

Prior to data collection, the study followed VID university college Institutional Review Board’s institutions ethical guidelines [35]. According to VID’s guidelines, studies that do not involve patient data are required to obtain approval from SIKT- Norwegian Agency for Shared Services in Education and Research [36]. SIKT serves as an institutional review body and is a knowledge sector service provider organized as a Norwegian governmental administrative body under the Ministry of Education and Research to assess whether privacy is adequately protected [36]. SIKT assessed the processing of personal data as lawful and approved the interview guide and informed consent. Before each interview, all participants provided written informed consent. During the study process, all data were anonymized by removing names, locations, and other identifying information. The interview transcription and audio files were securely stored in locked files. The research was conducted in accordance with the ethical principles outlined in the Declaration of Helsinki (2025). This article used the language model GPT-4o mini [37] for accurate translations from Norwegian to English. This tool was employed to ensure the correctness of the text and greater linguistic clarity [37].

## Discussion

This study explores HCNs’ experiences in facilitating home deaths for older patients. The findings indicate that older patients often have complicated health conditions, making it difficult to plan for a safe home death. In contrast to younger patients with cancer, nurses found that older patients frequently underwent unpredictable health changes, leading to many acute hospital admissions.

Nurses reported a lack of resources and varying quality of healthcare services, which hindered the possibility of a home death. They also expressed a need for security and support from GPs, which could be crucial in critical situations.

### A complex course of illness

A notable finding in this study is that nurses often perceive older patients as having complex and unpredictable illness trajectories. This is further corroborated by Huang et al. [26], who identified that patients with multimorbidity are usually particularly vulnerable and have significant care needs for which they do not always receive adequate support. In this study, nurses reported challenges in planning care when patients’ conditions were constantly changing. Huang et al. [26] also found that for older patients with multimorbidity, many hospital admissions could have been avoided. The complex illness trajectories of older patients pose significant barriers to their desire for a home death. The concept of ‘revolving-door patients’ is prominent in the findings, where nurses describe the chaotic pattern of frequent hospital admissions for older patients. According to Nicholson et al. [27], older patients with multimorbidity are less likely to receive palliative care, which can result in unnecessary and invasive treatments. Changes in health often initiate a transitional process in the patient’s life, rendering them more vulnerable to risks that may further impact their health [35].

Some nurses in this study expressed a need for the opportunity and resources to detect early signs of illness, potentially averting unnecessary hospital admissions.

In addition, nurses reported positive emotions when achieving a home death for a patient who desired it. Sørstrøm et al. [16] found similar outcomes; despite facing challenges, nurses felt a strong collective responsibility and commitment to facilitating home deaths for those patients who wished for it.

HCNs often work alone, without opportunities to discuss complex challenges with others [8]. In Norway, one of the primary responsibilities of HCNs is providing high-quality palliative care to patients in their homes [16]. Participants in this study expressed lack of experience and competence to provide palliative care to patients at home. The nurses called for more systematic care planning for older patients, which is crucial for quality care. They often compared older patients’ courses with younger cancer patients, noting that while the latter’s trajectories were usually well-planned, the formers were seemingly random and not discussed or planned. In this context, the concept of age discrimination is relevant, as Aminu et al. [25] suggest a correlation between age discrimination and the development of frailty. Similarly, Kjellstadli et al. [17] found that younger patients with cancer diagnoses more frequently die at home and that they that also engage more often in advanced care planning, as cancer diagnoses tend to be more predictable regarding their course of illness.

Meanwhile, Schumacher et al. [36] describe effective transitions in healthcare as processes that enable patients to gain control over their situations, promoting mastery and clarity. In contrast, the nurses in this study experienced that transitions for older patients were often chaotic, particularly for frequent users of healthcare services (“revolving door” patients). From the nurses’ perspective, these disordered transitions significantly limited their ability to manage care effectively and often led to a reduced sense of control over the care process. [36].

### Organizational inequalities

A key finding in this study is that older patients received different levels of care depending on where they lived. Significant organizational disparities also emerged between different municipalities in this study. Aminu et al. [25] point out that older patients may experience considerable delays in acute illnesses, and serious diagnoses can be overlooked due to potential age discrimination from healthcare providers. This aligns with the findings in this study, where all nurses experienced that older patients often followed a revolving door patient trajectory; their health courses were poorly planned.

A specific challenge that emerged in this study regarding the organization of services was the substantial disparities in availability between municipalities, particularly concerning night and standby resources when facilitating home deaths for older patients. In one municipality, a nurse could be hired for as long as the patient needed – days or weeks – while in another municipality, the home health care had to know it was the last night. This is supported by Sørstrøm et al. [16], who found that insufficient nursing resources significantly impacted the feasibility of home deaths. Another critical finding in Sørstrøm et al. [16] concerned the challenges associated with the night shift: there was fragmented collaboration between the day and night shifts, affecting nurses, patients, and relatives alike, as they did not know the night nurses, which could undermine the sense of security in the home. The present study similarly highlighted security as essential; achieving a home death was challenging without adequate security.

### The significant role of general practitioners

A notable finding was that the GP played a crucial role when patients wished to die at home. Without the GP, nurses experienced insecurity when dealing with complex challenges. During the evenings and weekends, when the GP was unavailable, only the emergency medical service could assist, and those workers were often unfamiliar with the patient, frequently resulting in unnecessary hospital admissions. Nurses indicated that they felt increased security when the GP was closely involved and sometimes provided their number for evenings and weekends, which often prevented superfluous hospital admissions at the end of life. Gjellestad et al. [19] found that there were unclear and weak structures for collaboration and clarification regarding who was responsible for what between GPs and HCNs. Although their study specifically focuses on patients with dementia, its findings align with how nurses in this study experienced cooperation with GPs. Ervik et al. [18] also found that GPs required increased competence in palliative care to ensure that it was not coincidental for patients whether they had a GP capable of providing good palliative care at the end of life.

### The critical role of relatives

The current study emphasizes that relatives played a critical role in facilitating home death. This is also supported by Sørstrøm et al. [16], who highlight the significant impact of relatives on home death, positively affecting patients in terms of quality of life but also presenting various negative aspects for next of kin (NOK). This aligns with findings from the current study, where the nurses experienced that NOK found home death to be emotionally challenging, alongside various difficulties. By the time HCNs became involved, NOK were often already experiencing fatigue. The nurses in this study expressed that they could not envision a successful home death occurring without deep involvement from the relatives.

## Strengths and limitations

One of the study’s main strengths is its qualitative approach. By utilizing interviews with home care nurses, the researchers uncovered nuanced perspectives and deep emotions related to the nurses past experiences with patients wishing to die at home. This method provides a comprehensive understanding that is not always captured by quantitative research. Another strength is that the participants come from four different municipalities in Norway, which allows for insights that reflect variations in different environments and approaches to home healthcare.

The first author’s experience and knowledge in home healthcare can be considered as both a strength and a limitation in this study – a strength in knowing the field and being able to ask relevant questions, and a weakness in that preconceptions can take things for granted and close one’s eyes to other possibilities. Throughout the research process, efforts were made to engage critically with this preunderstanding. Special attention was given in the interview, analysis, and writing phases to mitigate potential biases. The first author and the co-author had many rounds of reflection regarding preunderstanding, the analysis process, and the findings. The first author’s prior knowledge may facilitate a deeper comprehension of the conditions under which nurses operate in home care settings.

Another limitation of this study is that all participants were recruited from a single country with a specific healthcare system, which may limit the transferability of the findings to other contexts. Despite this, the study offers valuable insights into how home healthcare nurses understand and fulfil their roles in assisting elderly adults who wish to live and die at home. Indeed, to the authors’ knowledge, this is one of the few studies examining nurses’ experiences in facilitating home death for the elderly who wish for it. This unique focus can be viewed as a strength, highlighting that, despite its limited scope, the study makes a valuable contribution to the literature on this critical topic.

## Recommendations for further research

This study’s findings underscore the vital role of nurses in home healthcare. There, they frequently operate independently and are responsible for making critical decisions for elderly patients with complex health conditions. Further research is needed to understand these nurses’ challenges in delivering optimal care and support to older patients.

Moreover, investigating new systems and approaches to enhance these services could be highly advantageous. This study highlights a significant gap in research that specifically focuses on older patients living at home. While the study’s scope is limited, the insights gathered from participants provide valuable reflections of the realities faced in home nursing care. These realities further emphasize the pressing need for additional research and the development of innovative and improved systems to assist home healthcare nurses in delivering high-quality care to the elderly residing at home. Furthermore, Kjellstadli et al. [6] call for additional research on home deaths to assess whether patients’ places of death align with their actual preferences.

## Implications for policy and practice

It is essential to recognize the challenges that nurses face when facilitating home death for older patients who wish for it. This study’s findings provide valuable insights into the realities of home nursing and highlight the need for greater collaboration among policymakers, educators, and healthcare organizations. By working together, these stakeholders can establish better frameworks, ensure equitable services across different municipalities, and offer increased support for nurses, enabling them to navigate these demanding situations more effectively.

An early implementation of advanced care planning is crucial to allow more individuals to choose to die at home if they wish. This process is vital for older patients receiving home nursing care, as it provides opportunities for early discussions and clarifications, extending this practice beyond younger patients with serious illnesses. Furthermore, enhancing the competencies of HCNs is vital to address the growing number of older adults in the coming years. In support of this need, Sørstrøm et al. [20] emphasize the demand for more knowledge regarding how HCNs deliver end-of-life care. By prioritizing these areas, nurses can be better equipped to manage the complexities of their roles and ultimately improve the quality of care for older patients in home settings.

## Conclusion

This study highlights nurses’ difficulties in facilitating home deaths, revealing barriers such as organizational inconsistencies and limited resources. Addressing these challenges is crucial for improving palliative care and support for older patients. Insights from this study can guide decision-makers in considering enhancements to home healthcare services. Given the rapidly aging global population, the findings of this research will significantly contribute to highlighting the challenges nurses face in home care services in Norway and worldwide.

## Data Availability

The datasets generated and analysed during the current study are not publicly available due to privacy considerations. However, they are available from the corresponding author on reasonable request.

## Abbreviations

HCN: Home care nurses
GP: General practitioner
NOK: Next of kind
